# Treatment Challenges in Alopecia Areata: Insights From a Case of Baricitinib Therapy Failure

**DOI:** 10.7759/cureus.63120

**Published:** 2024-06-25

**Authors:** Deesha D Desai, Ambika Nohria, Kristen I Lo Sicco, Jerry Shapiro

**Affiliations:** 1 Department of Dermatology, University of Pittsburgh School of Medicine, Pittsburgh, USA; 2 The Ronald O. Perelman Department of Dermatology, NYU Grossman School of Medicine, New York, USA

**Keywords:** clinical case, hair loss, baricitinib, alopecia areata, alopecia

## Abstract

Alopecia areata (AA) has long been considered a challenging clinical condition, with dermatologists traditionally employing corticosteroids and immunosuppressants in search of effective solutions. The introduction of Janus kinase inhibitors (JAKi), specifically the Food and Drug Administration (FDA) approval of baricitinib, marked a significant breakthrough in the treatment of AA. Clinical trials have shown promising results with baricitinib, and reports of relapse after initial success are scarce. We present a unique case of a 30-year-old male with severe patch-type AA who initially responded well to baricitinib treatment but later experienced a relapse despite continued treatment.

## Introduction

The approval of baricitinib by the Food and Drug Administration (FDA) in June 2022 marked a significant milestone in the management of alopecia areata (AA), establishing it as the first official on-label therapy for this condition [1]. This marked a transformative shift in the therapeutic landscape of AA, which had previously relied on off-label therapies such as corticosteroids and immunosuppressants. Before the introduction of baricitinib, there had been sparse data documenting instances of treatment failures associated with Janus kinase inhibitors (JAKi) such as ruxolitinib [2]. Remarkably, despite the growing body of evidence supporting the efficacy of JAKi in AA, including promising outcomes with baricitinib, there is a lack of data regarding non-responders to this medication. We present a unique case involving a patient diagnosed with severe patch-type AA, who initially exhibited a positive response to baricitinib therapy but later experienced a relapse despite ongoing treatment.

## Case presentation

A 30-year-old healthy male initially sought treatment at the New York University (NYU) hair clinic due to patchy hair loss on his scalp and beard for one year. The physical exam revealed widespread, patchy hair loss affecting many areas, including the occipital, vertex, and parietal regions of his scalp, along with his beard with no nail involvement. Trichoscopic analysis revealed yellow dots and exclamation mark hairs, leading to a diagnosis of severe patch-type AA. The initial treatment involved nine rounds of monthly intralesional triamcinolone (IL-TAC) scalp injections. This approach led to minimal hair regrowth on the scalp but no regrowth on the beard. Afterward, the patient was also started on low-dose oral minoxidil (LDOM) (2.5 mg daily) and topical ruxolitinib (1.5% cream). Despite this additional therapy, the progress remained limited, even after a total of 16 rounds of IL-TAC injections.

**Figure 1:**
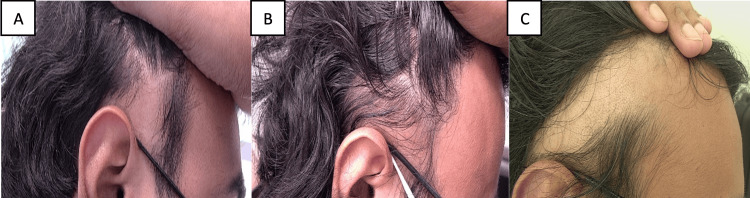
Patient images - 1 A. Before baricitinib therapy. B. Six months into baricitinib therapy. C. 13 months into baricitinib therapy

**Figure 2:**
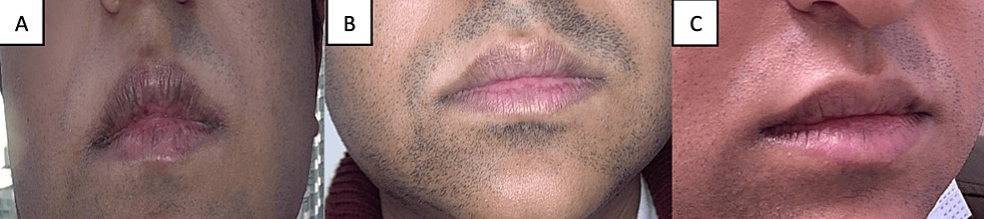
Patient images - 2 A. Before baricitinib therapy. B. Six months into baricitinib therapy. C. 13 months into baricitinib therapy

In August 2022, the patient was noted to have widespread scalp involvement of greater than 50%. At this time, the treatment plan was modified, and the patient began oral baricitinib 2 mg daily and continued with LDOM 2.5 mg daily. After one month, the baricitinib dosage was increased to 4 mg daily and the LDOM 2.5 mg daily was continued. By the end of the third month on baricitinib, the patient reported significantly less hair shedding, along with marked regrowth of his beard, mustache, and scalp. This positive progress continued for the next six months. However, after these nine months of improvement, the patient experienced a resurgence of severe patchy hair loss, with new patches appearing despite ongoing treatment and lack of any potential stressful triggers. Given these developments, and after close monitoring for the next four months, the patient was deemed to have failed baricitinib therapy for 13 months and was switched to ritlecitinib thereafter (Figures 1A-1C, Figure 2A-2C). Unfortunately, even after five months on ritlecitinib, the patient has not shown any signs of improvement and continues to develop new patches.

## Discussion

AA is a form of non-scarring alopecia with significant psychosocial ramifications [3]. While this condition can often resolve on its own, severe cases, such as our patients' patch-type AA, with a spontaneous remission rate of only 30-50% within the first 6-12 months have also been reported [4]. Recent advances in clinical research have provided valuable insights into the underlying pathophysiology of AA, leading to the emergence of new treatment approaches. Notably, the discovery of the JAK-STAT pathway’s pivotal role in this condition has prompted the introduction of JAKi as a new treatment modality [5,6]. Among these, baricitinib, a selective JAK1 and JAK2 inhibitor, has emerged as a promising therapy option for individuals with severe AA. Clinical trials have demonstrated remarkable efficacy. Specifically, among patients treated with baricitinib 4 mg and 2 mg, respectively, 40.9% and 21.2% in BRAVE-AA1 and 36.8% and 24.4% in BRAVE-AA2 achieved a Severity of Alopecia Tool (SALT) score ≤20 at Week 52 [7].

Despite the promising outcomes observed with baricitinib, the unpredictable nature of AA and a serious concern for its relapse underscore the importance of maintaining treatment regimens. While previous reports have documented relapses in patients treated with ruxolitinib, no such instances have been reported with baricitinib so far [2]. In this context, we presented a case of a patient who experienced a relapse of his AA despite ongoing treatment with baricitinib and LDOM. This case raises concerns regarding the potential for relapse in patients while on baricitinib therapy. Further research is imperative to comprehensively evaluate the efficacy of baricitinib in long-term AA management, understand the duration necessary to determine if the treatment has failed, and assess if LDOM decreases the risk of relapse.

## Conclusions

This case report highlights the complexities and uncertainties inherent in treating AA. While baricitinib has demonstrated significant efficacy in clinical trials, the relapse experienced by our patient underscores the need for continuous monitoring and adaptability in treatment approaches. Further research is crucial to better understand the underlying factors contributing to the failure of baricitinib, as well as explore alternative treatment options.

## References

[1] (2024). Olumiant (baricitinib) FAQ - National Alopecia Areata Foundation. https://www.naaf.org/olumiant-baricitinib-faq/.

[2] Deeb M, Beach RA (2017). A case of topical ruxolitinib treatment failure in alopecia areata. J Cutan Med Surg.

[3] Toussi A, Barton VR, Le ST, Agbai ON, Kiuru M (2021). Psychosocial and psychiatric comorbidities and health-related quality of life in alopecia areata: a systematic review. J Am Acad Dermatol.

[4] Alhanshali L, Buontempo MG, Lo Sicco KI, Shapiro J (2023). Alopecia areata: burden of disease, approach to treatment, and current unmet needs. Clin Cosmet Investig Dermatol.

[5] Lensing M, Jabbari A (2022). An overview of JAK/STAT pathways and JAK inhibition in alopecia areata. Front Immunol.

[6] Singla S, Narang R, Shanker V, Gupta S, Saraswat N, Singh R (2023). Analyzing the role of tofacitinib in treatment of alopecia areata: a retrospective analysis from a tertiary care center of North India. Natl J Physiol Pharm Pharmacol.

[7] Kwon O, Senna MM, Sinclair R (2023). Efficacy and safety of baricitinib in patients with severe alopecia areata over 52 weeks of continuous therapy in two phase III trials (BRAVE-AA1 and BRAVE-AA2). Am J Clin Dermatol.

